# Draft Genome Sequence of *Shewanella* sp. Strain P1-14-1, a Bacterial Inducer of Settlement and Morphogenesis in Larvae of the Marine Hydroid *Hydractinia echinata*

**DOI:** 10.1128/genomeA.00003-16

**Published:** 2016-02-18

**Authors:** Maja Rischer, Jonathan L. Klassen, Thomas Wolf, Huijuan Guo, Ekaterina Shelest, Jon Clardy, Christine Beemelmanns

**Affiliations:** a Leibniz Institute for Natural Product Research and Infection Biology e.V., Jena, Germany; b Department of Molecular & Cell Biology, University of Connecticut, Storrs, Connecticut, USA; c Department of Biological Chemistry and Molecular Pharmacology, Harvard Medical School, Boston, Massachusetts, USA

## Abstract

The assembly and annotation of the draft genome sequence of *Shewanella* sp. strain P1-14-1 are reported here to investigate the genes responsible for interkingdom interactions, secondary metabolite production, and microbial electrogenesis.

## GENOME ANNOUNCEMENT

Members of the genus *Shewanella* are facultative anaerobic bacteria colonizing mostly marine and freshwater environments (1, 2) and have been shown to be metal-reducing bacteria capable of transferring electrons to the environment by different mechanisms (3). Besides their bioremediation capabilities and electrogenic potential, *Shewanella* spp. are known to produce secondary metabolites and induce settlement in larvae of biofouling marine invertebrates (4, 5). In our quest to isolate and characterize chemical cues involved in the larval settlement of *Hydractinia echinata* (6) and to identify the responsible genes and regulation mechanisms, we isolated, assessed, and sequenced marine microbes commonly associated with *H. echinata* (7, 8). *Shewanella* sp. strain P1-14-1 was isolated from the tissue from a feeding polyp of the marine hydroid *H. echinata*, purchased from the Marine Biological Laboratory in Woods Hole, MA. We first tested larval settlement and morphogenic activity using a larva-based assay, and then its antimicrobial activity was assessed. *Shewanella* sp. P1-14-1 showed high and reliable morphogenic activity, with a metamorphosis rate of up to 80%, and moderate antibacterial activity against Gram-positive human-pathogenic bacteria. To investigate the genetic basis of potential antimicrobial secondary metabolites (9) and the molecular details of the morphogenic activity, the genome was sequenced. Genomic DNA was extracted using the GenElute blood genomic DNA kit (Sigma-Aldrich), according to the manufacturer’s protocol. Sequencing was performed at the Harvard Medical School Biopolymers Facility using Illumina TruSeq 50-bp paired-end libraries and a HiSeq 2000 flow cell (CASAVA 1.8.2; Illumina). DNA sequence data were assembled using the A5 pipeline version 20120518 (10) and analyzed for potential contaminations using Blobology (11). Genome annotation was performed using Prokka version 1.8 (12), and the G+C content was determined using QUAST version 3.0 (13). The draft genome of P1-14-1 is 4,916,416 bases in length; the G+C content is 40.78%, with a total of 150 contigs. The largest contig assembled was 301,078 bp. The total coverage of the genome 2is 46-fold. Genome annotation resulted in 4,159 coding sequences (CDSs), 75 tRNAs, and 5 rRNAs. Genes associated with biofilm formation and surface attachment, including genes encoding flagella, curli, type II secretion system, type IV pili, and capsular polysaccharide (O-antigen) proteins were identified, reflecting the adaptation to successful persistence and competition on aquatic surfaces (14). The production of pili and flagella has also been connected with enhanced electrogenesis in bacteria (15, 16). Genes encoding bacteriocins and secondary metabolites (e.g., antibiotic pyrrolnitrin), were detected using antiSMASH (17) and SMIPS (18).

The draft genome sequence of *Shewanella* sp. P1-14-1 will promote the genetic analysis of the *Shewanella* genus and provide insights into secondary metabolite production and the genetic basis of the bacterial signals, which induce the settlement process of *H. echinata*.

### Nucleotide sequence accession numbers.

The *Shewanella* sp. P1-14-1 whole-genome shotgun (WGS) project has the project accession no. LKTL00000000. The version described in this paper is the first version, with the accession number LKTL01000000.
